# Regional gray matter correlates of vocational interests

**DOI:** 10.1186/1756-0500-5-242

**Published:** 2012-05-16

**Authors:** David H Schroeder, Richard J Haier, Cheuk Ying Tang

**Affiliations:** 1 Johnson O’Connor Research Foundation, Chicago, IL, USA; 2 University of California, School of Medicine (Emeritus), Irvine, CA, USA; 3 Mt. Sinai Medical Center, School of Medicine, New York City, USA

## Abstract

**Background:**

Previous studies have identified brain areas related to cognitive abilities and personality, respectively. In this exploratory study, we extend the application of modern neuroimaging techniques to another area of individual differences, vocational interests, and relate the results to an earlier study of cognitive abilities salient for vocations.

**Findings:**

First, we examined the psychometric relationships between vocational interests and abilities in a large sample. The primary relationships between those domains were between Investigative (scientific) interests and general intelligence and between Realistic (“blue-collar”) interests and spatial ability. Then, using MRI and voxel-based morphometry, we investigated the relationships between regional gray matter volume and vocational interests. Specific clusters of gray matter were found to be correlated with Investigative and Realistic interests. Overlap analyses indicated some common brain areas between the correlates of Investigative interests and general intelligence and between the correlates of Realistic interests and spatial ability.

**Conclusions:**

Two of six vocational-interest scales show substantial relationships with regional gray matter volume. The overlap between the brain correlates of these scales and cognitive-ability factors suggest there are relationships between individual differences in brain structure and vocations.

## Findings

A growing number of neuroimaging studies focus on individual differences in mental abilities. There are now many studies of the general factor of intelligence (*g*) (see reviews by Jung & Haier [1] and Deary et al. [2]) and specific intelligence factors (e.g., [3,4]). Jung and Haier [1] introduced the Parieto-Frontal Integration Theory, or P-FIT, specifically to account for the neuroimaging findings regarding general intelligence. There are also many imaging studies of personality (see review by Haier [5] and also DeYoung et al. [6]).

One area of individual differences that has not been studied with neuroimaging techniques is vocational interests. Individuals show wide variation on multiple dimensions of interests, and these differences have important implications for career choice [7]. Interests are also related to abilities [8] and to personality [9], and since those domains are related to brain differences, it is possible that vocational interests will also show meaningful relationships with the brain.

**Figure 1:**
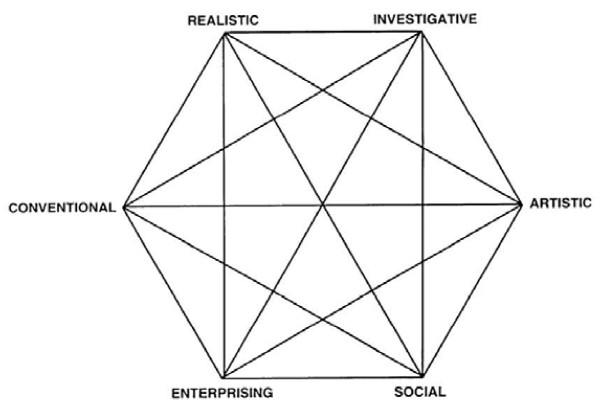
Holland’s hexagon model for vocational interests.

The most widely used and empirically studied model of vocational interests was developed by Holland [7]. As summarized in Table 1, Holland’s hexagon model incorporates six interest areas: Realistic, Investigative, Artistic, Social, Enterprising, and Conventional. As shown in Figure 1, the hexagon structure corresponds to the relationships among the six areas: Enterprising interests are most closely related to Social and Conventional interests and least related to Investigative interests, Realistic interests are most related to Conventional and Investigative interests and least related to Social interests, and so on.

**Table 1:** Scales on the self-directed search

**Scale**	**Reliability**	**Description**
Realistic	.92	Interest in outdoor, hands-on activities that involve tangible objects.
Investigative	.93	Interest in scientific work.
Artistic	.92	Interest in artistic endeavors of various types such as visual art, music, and writing.
Social	.92	Interest in social interaction, often in human-service contexts.
Enterprising	.93	Interest in social interaction in a context of selling and/or persuasion.
Conventional	.93	Interest in highly structured activities including clerical and administrative functions.

*Note*. The reliability coefficients are averages of the internal-consistency reliabilities for adult males and females, respectively, given in the SDS technical manual p. 22 [10].

Recently, we reported relationships between regional gray matter and mental abilities specifically related to vocations, along with white matter correlates, using a battery of tests developed by the Johnson O’Connor Research Foundation [11,12]. Here we report on gray matter correlates of vocational interests in the same sample. This allows a direct comparison of the gray matter correlates for vocational interests with the correlates for ability dimensions related to vocations.

Based on Holland’s model, we tested specific hypotheses regarding two of his scales:

1. Because vocational interests in the Investigative area are related to general intelligence (*g*) [13], we hypothesized that Investigative interests would show similar brain correlates to *g*[1].

2. Because vocational interests in the Realistic area are related to spatial ability [14], we hypothesized that Realistic interests would show similar brain correlates to spatial ability [3,4].

The four other areas of vocational interests (Artistic, Social, Enterprising, Conventional) have shown a lesser degree of relationship with cognitive abilities [8], and so we explored whether they would show any separate relationships with regional gray matter volume.

Like many neuroimaging studies, this one used a relatively small sample, and so it should be viewed as an exploratory investigation into the brain correlates of vocational interests. Because of the sample size, we did not examine sex differences.

## Analysis one: psychometric relationships

We started by examining the relationships between interests and abilities in a large sample before we looked at the neuroimaging data in a smaller subset of subjects.

### Ethics statement

Each participant gave written informed consent as approved by the Executive Committee of the Johnson O’Connor Research Foundation. This research was conducted in accord with the Helsinki Declaration.

### Subjects

During 2006–07, 8,181 individuals sought consultation from the Johnson O’Connor Research Foundation (JOCRF), a nonprofit organization dedicated to using psychometric assessments for vocational guidance. Each completed a battery that included eight cognitive-ability tests and a vocational-interest measure (see below) in one of 11 testing offices in major cities throughout the United States. The mean age for all subjects was 24.9 years (*SD* = 10.8); there were 4,438 males (mean age = 24.2, *SD* = 10.0) and 3,743 females (mean age = 25.8, *SD* = 11.6).

### Vocational-interest measure

We measured six vocational interest areas with Holland’s Self-Directed Search (SDS) [15]. As noted, these areas are described in Table 1 and the hexagon model shown in Figure 1.

### Cognitive-ability measures

Eight tests from the Johnson O’Connor battery were given: Inductive Speed (IS), Analytical Reasoning (AR), Number Series (NS), Number Facility (NF), Wiggly Block (WB), Paper Folding (PF), Verbal-Associative Memory (VAM), and Number Memory (NM). A description of these tests, including the constructs they measure and their reliabilities can be found in the Haier et al. article [4], along with confirmation that this battery loads on four group factors – Speed of Reasoning (IS and AR), Numerical (NS and NF), Spatial (WB and PF), and Memory (VAM and NM) in addition to a *g*-factor. The means and standard deviations for all of the tests are shown in Table 2.

**Table 2:** Descriptive statistics for samples

	**Study one (*N* = 8,181)**		**Study two (*N* = 40)**	
Measure	*M*	*SD*	*M*	*SD*
**Self-directed search**				
Realistic	20.03	10.30	19.65	7.96
Investigative	22.95	9.99	24.80	9.99
Artistic	23.71	10.79	25.88	9.22
Social	27.21	9.29	27.33	9.69
Enterprising	26.54	9.79	28.05	9.82
Conventional	18.75	8.75	22.15	6.56
**Cognitive abilities**				
*Spatial*				
Wiggly block	277.49	99.27	320.35	88.60
Paper folding	22.49	13.86	28.73	14.68
*Numerical*				
Number series	23.71	4.58	24.48	4.98
Number facility	94.79	17.34	100.66	19.38
*Memory*				
Verbal-associative memory	21.54	9.74	24.40	10.14
Number memory	81.72	28.68	91.55	28.17
*Speed of reasoning*				
Inductive speed	141.50	22.58	141.15	22.22
Analytical reasoning	54.83	12.79	60.32	14.20

*Note*. The reported values are for raw scores unpartialled for sex, age, or *g*. The Study Two sample scored significantly higher than the Study One sample on Wiggly Block, Paper Folding, Number Facility, Number Memory, and Analytical Reasoning (*p* < .05). The two groups did not differ significantly on any of the interest measures except for Conventional, on which the Study Two sample scored about .4 of a standard deviation higher than the Study One sample.

We computed standardized scores (*z*-scores) for the eight tests and computed average *z*-scores for each factor. The general intelligence *g*-score for each subject was the average of their *z*-scores on the eight tests (see Haier et al. [4] for additional details) with an alpha reliability of .80. The *g* and residualized (that is, *g*-partialled) *z*-scores for each factor were used to determine the correlations with the vocational interest scales. Note that residualized scores for speed of reasoning, numerical, spatial, and memory represent participants’ performance not shared with the general factor score (*g*). Test scores on both the interest and ability measures were partialled for sex and age in order to eliminate nuisance variance.

### Results

The correlations between the six SDS scales and the Johnson O’Connor ability factors are shown in Table 3. As can be seen, the correlations are generally modest. The largest correlation (*r* = .41) is between *g* and Investigative interests. There is also a moderate correlation (.30) between Realistic interests and the Spatial factor (again, with *g* partialled out). These results support our particular focus on brain areas related to both Realistic interests and Spatial ability and to Investigative interests and general ability.

**Table 3:** Correlations between SDS scales and JOCRF ability factors (N = 8,181)

**Ability factor**					
SDS scale	*g*	Spatial	Numerical	Memory	Speed of reasoning
Realistic	.14	.30	-.19	-.16	.04
Investigative	.41	.07	.03	-.05	-.06
Artistic	.08	.02	-.12	.05	.04
Social	-.03	-.14	.05	.05	.05
Enterprising	.01	-.11	.09	.01	.02
Conventional	.15	-.07	.16	.01	-.08

*Note*. All correlations greater than .02 (or less than -.02) are significant at the *p* = .01 level. For the ability factors, each factor other than *g* was partialled for *g*.

## Analysis two: imaging

### Ethics statement

In addition to the JOCRF consent described above, each participant for this portion of the study gave written informed consent for the imaging research described below, as approved by the Mt. Sinai Medical Center Institutional Review Board. This research was conducted in accord with the Helsinki Declaration.

### Subjects

The subjects are a subset of those in the above analysis and the same as those described in several previous reports [4,11,12]. Subjects between the ages of 18 and 35 who completed the test battery in the New York City office were invited to return for MRI scanning at Mt. Sinai Medical Center. All who volunteered were screened for medical and psychiatric illnesses including a history of head injury and substance abuse. 40 subjects consented and completed MRI (21 males and 19 females, with a mean age of 26.6, *SD* = 4.9). They all completed the same battery of ability tests and the SDS, as described above.

The means and standard deviations for this sample on the interest and ability scales are shown in Table 2. The distributions for this sample are fairly similar to the distributions for the larger JOCRF sample. The means are somewhat higher than for the larger sample on the ability scales, but the standard deviations are similar, which indicates sufficient variation to support correlation analyses.

### Structural MRI acquisition

A 3T Siemens Allegra MRI scanner (Siemens Medical Systems, Erlangen, Germany) was used at Mt. Sinai Medical Center, NYC. For each subject, a sagittal T_1_-weighted spin echo image was performed first as localizer. Based on this localizer, structural scans were acquired using a 3D MP-RAGE pulse sequence with the following parameters: TR = 2500 ms, TE = 4.4 ms, FOV = 21 cm, matrix size = 256 × 256, 208 slices with thickness = 0.82 mm.

### Voxel-based-morphometry (VBM) and statistical analyses

Using Statistical Parametric Mapping software (SPM5; The Wellcome Department of Imaging Neuroscience, University College London), we applied voxel-based morphometry (VBM) to identify brain areas where gray matter (GM) volumes are correlated with test scores. The structural images were bias field corrected, and segmented using an integrated generative model (unified segmentation, [16]). Unified segmentation involves alternating between segmentation, bias field correction, and normalization to obtain local optimal solutions for each process. The default SPM5 tissue probability maps were used (tissue probability maps provided by the International Consortium for Brain Mapping T1 452 Atlas [17]). The final segmentations were modulated [18] and GM partitions were smoothed with a 12-mm FWHM isotropic Gaussian kernel to account for slight misalignments of homologous anatomical structures and to ensure statistical validity under parametric assumptions.

**Figure 2:**
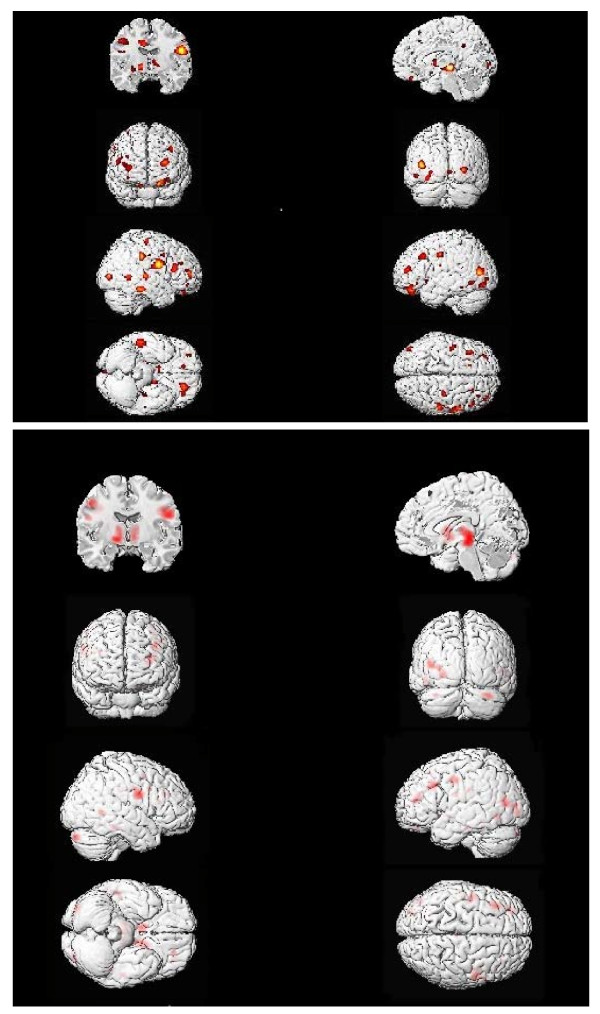
**Gray matter correlates of Investigative interests and overlap of correlates of Investigative and*g.*** Gray matter correlates (positive) of Investigative interest scores (top panel), *N* = 40 (*p* < .01 yellow, *p* < .025 orange, *p* < .05 red); and overlap of Investigative and JOCRF *g* correlates (bottom panel; *p* < .001 FWE).

**Figure 3:**
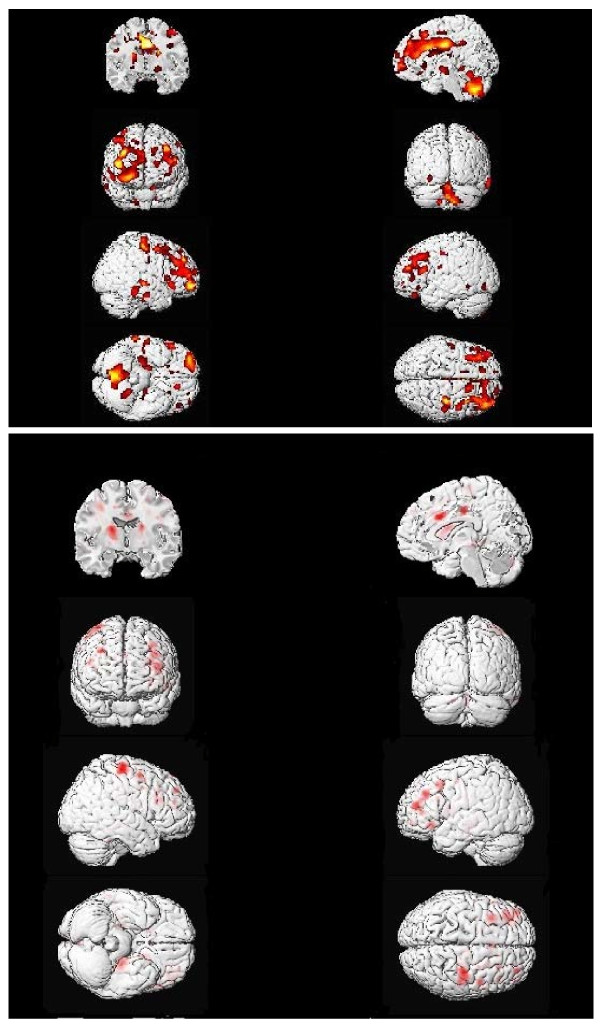
**Gray matter correlates of Realistic interests and overlap of correlates of Realistic and spatial ability.** Gray matter correlates (positive) of Realistic interest scores (top panel), *N* = 40 (*p* < .01 yellow, *p* < .025 orange, *p* < .05 red); and overlap of Realistic and JOCRF Spatial correlates (bottom panel; *p* < .001 FWE).

To identify areas where GM correlated to SDS scales, we used a significance level of *p* < .001 uncorrected in view of the small sample size and the exploratory nature of this study. To test the two hypotheses, we determined overlap between I and the *g*-factor, and between R and the spatial factor, as described previously [4] using the *xj*-view tool from SPM5, with *p* < .05 for each scale; we report only overlap areas significant at *p* < .001 corrected using FWE (family wise error). Xjview overlays multiple statistical images on top of each other to show statistical overlap for the images. If each of two analyses is thresholded at *p* < .05 uncorrected, the resulting image will have a threshold which is multiplicative. Thus *p* < .05 X *p* < .05 = *p* < .0025. The result is a conjunction of the analyses, or the probability of the independent results overlapping in a particular brain region. For Tables 4 and 5 and Figures 2 and 3, the probability of the correlates of interest overlapping in the brain regions indicated reaches the FWE threshold of *p* < .001 even though each individual analysis does not reach that threshold, so more significant regions are identified. Also, more regions appear significant at *p* < .001 FWE corrected in Table 5 because the areas covered by the overlap are smaller (fewer voxels) and thus one region which covered a large area in the original analysis is broken up into multiple regions.

**Table 4:** Gray matter correlates of investigative Interest scores and overlap of investigative and JOCRF g correlates (N = 40)

**Size**	***p***	***x,y,z***				
*Investigative* (*p* < .001, unc.)						
384	0.000	52 0 24	Right	Frontal	Inferior frontal gyrus	Brodmann area 9
204	0.000	-42 -74 12	Left	Temporal	Middle temporal gyrus	Brodmann area 39
190	0.000	-26 48 14	Left	Frontal	Middle frontal gyrus	Brodmann area 10
177	0.001	54–32 -18	Right	Temporal	Inferior temporal gyrus	Brodmann area 20
110	0.001	-14 -6 -2	Left	Sub-lobar	Lentiform nucleus	Medial globus pallidus
585	0.001	-38 -44 16	Left	Sub-lobar	Insula	Brodmann area 13
147	0.001	-40 -10 40	Left	Frontal	Precentral gyrus	Brodmann area 6
112	0.001	30–84 4	Right	Occipital	Middle occipital gyrus	Brodmann area 19
100	0.001	48–52 2	Right	Temporal	Middle temporal gyrus	Brodmann area 37
*Overlap of Investigative and* g (*p* < .001, FWE-cor.)						
263	0.000	-36 22 36	Left	Frontal	Middle frontal gyrus	Brodmann area 9
448	0.000	52 0 26	Right	Frontal	Inferior frontal gyrus	Brodmann area 9
434	0.000	-42 -74 12	Left	Temporal	Middle temporal gyrus	Brodmann area 39
579	0.000	-14 -6 -4	Left	Sub-lobar	Lentiform nucleus	Medial globus pallidus
1081	0.000	12–22 -8	Right	Midbrain	--	Substania nigra
240	0.000	-28 46 14	Left	Frontal	Middle frontal gyrus	Brodmann area 10
76	0.000	-48 -60 -6	Left	Temporal	Inferior temporal gyrus	Brodmann area 19

*Note*. All correlations are positive; size is number of voxels in significant cluster; *p* for Investigative correlates is uncorrected; *p* for overlap correlates is FWE-corrected; *x,y,z* coordinates are maximum voxel (MNI); left/right is hemisphere; Brodmann areas are best estimates from Talairach and Tournoux Atlas (1988).

**Table 5:** Gray matter correlates of realistic interest scores and overlap of realistic and JOCRF spatial correlates (N = 40)

**Size**	***p***	***x,y,z***				
*Realistic* (*p* < .001, unc.)						
7031	0.000	8–14 34	Right	Limbic	Cingulate gyrus	Brodmann area 23
1177	0.000	-34 20 40	Left	Frontal	Precentral gyrus	Brodmann area 9
300	0.000	-30 -22 40	Left	Parietal	Postcentral gyrus	Brodmann area 3
1142	0.000	72–36 -18	Right	Temporal	Middle temporal gyrus	Brodmann area 21
100	0.000	-18 48 14	Left	Frontal	Medial frontal gyrus	Brodmann area 10
210	0.001	-28 -76 -6	Left	Occipital	Lingual gyrus	Brodmann area 19
802	0.001	34–18 46	Right	Frontal	Precentral gyrus	Brodmann area 4
255	0.001	64 20 4	Right	Frontal	Inferior frontal gyrus	Brodmann area 45
2479	0.001	8–70 -38	Right	Posterior	Uvula	--
283	0.001	-20 10 14	Left	Sub-lobar	Lentiform nucleus	Putamen
*Overlap of Realistic and Spatial factor* (*p* < .001, FWE-cor.)						
206	0.000	-32 14 42	Left	Frontal	Middle frontal gyrus	Brodmann area 8
147	0.000	8–16 36	Right	Limbic	Cingulate gyrus	Brodmann area 24
423	0.000	-18 6 14	Left	Sub-lobar	Lentiform nucleus	Putamen
179	0.000	22 2 18	Right	Sub-lobar	Lentiform nucleus	Putamen
479	0.000	-28 -22 -6	Left	Sub-lobar	Lentiform nucleus	Putamen
359	0.000	36–20 70	Right	Frontal	Precentral gyrus	Brodmann area 6
85	0.000	30 24 38	Right	Frontal	Middle frontal gyrus	Brodmann area 9
160	0.000	6 16 30	Right	Limbic	Cingulate gyrus	Brodmann area 24
54	0.000	34 52 38	Right	Frontal	Superior frontal gyrus	Brodmann area 9
77	0.001	-56 28–10	Left	Frontal	Inferior frontal gyrus	Brodmann area 47
600	0.001	-36 34 34	Left	Frontal	Superior frontal gyrus	Brodmann area 9

*Note*. All correlations are positive; size is number of voxels in significant cluster; *p* for Realistic is uncorrected; *p* for overlap correlates is FWE-corrected; *x,y,z* coordinates are maximum voxel (MNI); left/right is hemisphere; Brodmann areas are best estimates from Talairach and Tournoux Atlas (1988).

### Results

Positive GM correlates of the Investigative (I) scale are shown in Figure 2 (top) and detailed in Table 4 (*p* < .001 uncorrected). Nine areas were found including large clusters in Brodmann Areas (BA) 9, 10, 13, 39 and the globus pallidus. There were two small clusters where the correlations were negative (BA 7 and 38; with only 82 and 27 voxels, respectively). None of these correlations survived FWE correction.

The overlap of these areas with areas related to *g* is shown in the bottom of Figure 2 and detailed in Table 4. Using FWE corrected values at *p* < .001, the main overlap areas were BA 9, 39 and the globus pallidus.

GM correlates of the Realistic (R) scale are shown in Figure 3 (top panel) and detailed in Table 5 (*p* < .001, uncorrected). GM is positively correlated with R in a number of brain areas. In particular, there is a large cluster that includes portions of the cingulate gyrus in BA 23, and there are good-sized clusters in BA 4, 9, 21, and the uvula lobe in the cerebellum. The overlap with the spatial factor is shown in the bottom panel of Figure 3 and detailed in Table 5. Main overlap areas include BA 8, 24, and the putamen.

To help visualize the overlap results, Additional file 1: Figure S1 and Additional file 2: Figure S2 show the VBM correlates of the interest scales and the cognitive-ability scores, both at *p* < .05.

In contrast, there were only a few small sporadic GM correlates of the other four interest scales at *p* < .001 uncorrected (not shown).

## Discussion

As noted, our intention in this exploratory study is to introduce imaging techniques to the study of individual differences in vocational interests. Our findings from Analysis One demonstrate there are associations between two interest scales (I and R) and two cognitive abilities (*g* and spatial factors). Furthermore, Analysis Two shows that, as hypothesized, there is overlap between GM in the respective brain areas for I and R interests and in areas related to the *g* and spatial factors, respectively. Such relationships have not been identified previously.

It may be the case that I and R interests lead persons to invest time and effort into pursuits that benefit general intelligence and spatial ability, respectively. For example, a boy with realistic interests may play with Lincoln Logs and build model airplanes, and these activities may foster the development of visualization ability in a manner akin to the development of crystallized intelligence in Cattell’s investment theory [19]. On the other hand, it is possible that spatial ability and general intelligence lead to positive experiences with outdoor and scientific endeavors, which then lead to interests in Realistic and Investigative areas, respectively [7].

Although our data do not speak to the direction of effect, the Investigative and Realistic interest scales were the two that showed relationships with brain structure. Because of the small sample size, results based on VBM must not be overinterpreted, so we are only commenting on results relevant to our hypotheses regarding overlap of brain areas for these interest scales and the two ability factors that reach significance with FWE correction. The I scale overlap with the *g*-factor (Table 4) is mostly in prefrontal association (BA 9, 10) and parietal/temporal areas (BA 39) and the substania nigra. All but the latter have been associated with general intelligence previously across several studies [1]. The R scale overlap with the spatial factor (Table 5) is mostly in frontal areas (BA 6, 8, 9, 47) along with the putamen and a part of the cingulate gyrus. Although spatial ability is often associated with the right hemisphere, we find areas in both hemispheres, possibly due to idiosyncrasies in our sample. Note also that this set of overlap areas is larger than those associated with just spatial ability here and in our 2009 [4] and 2010 papers [11] because of the multiplicative nature of the xj view analysis as explained earlier.

Our data suggest that individual differences in I and R vocational interests may be attributable to brain differences in areas related to general intelligence and spatial ability, respectively. For the other four vocational-interest scales, there was little relationship with cognitive-ability factors or brain structure. Interests in those areas may be influenced by temperament factors [7], which were not studied here, but if that is the case, one might have expected to see relationships with brain areas associated with temperament [6]. On the other hand, interests in these areas may be largely due to socialization and life experience, and these influences may be relatively independent of brain structure as measured here.

Finding brain correlates of any psychometric scale provides some validity for the scale. Our results suggest that some but not all vocational interests may reflect brain characteristics. How a characteristic like gray matter volume may develop, and how it may lead to individual differences related to vocations, is far from clear. This study demonstrates that neuro-imaging may help provide clues to these fundamental questions.

## Competing interests

This project was funded at the Mt. Sinai Medical Center (Cheuk Tang), New York City, by the nonprofit Johnson O’Connor Research Support Corporation (JOCRSC). Cheuk Tang received partial salary support, and Richard Haier is a paid consultant of the JOCRSC. David Schroeder is an employee of the Johnson O’Connor Research Foundation (JOCRF), which receives financial support from the JOCRSC. The JOCRSC did not have any approval or supervisory role in the preparation of the manuscript or the decision to publish.

## Authors’ contributions

RJH and DHS conceived and designed the study. CYT collected the imaging data. DHS and RJH analyzed the data. All authors approved and helped write the manuscript.

## Supplementary Material

Additional file 1 **Figure S1.** Gray matter correlates of Investigative interest scores (red) and JOCRF *g* scores (blue), both shown at *p* < .025. Overlap is purple. (JPEG 455 kb) Click for file here

Additional file 2 **Figure S2.** Gray matter correlates of Realistic interest scores (red) and JOCRF Spatial scores (blue), both shown at *p* < .025. Overlap is purple. (JPEG 460 kb) Click for file here

## References

[B1] Jung RE Haier RJ The Parieto-Frontal Integration Theory (P-FIT) of intelligence: Converging neuroimaging evidence Behav Brain Sci 2007 30 135 154 10.1017/S0140525X07001185 17655784

[B2] Deary IJ Penke L Johnson W The neuroscience of human intelligence differences Nat Rev Neurosci 2010 11 201 211 20145623 10.1038/nrn2793

[B3] Colom R Haier RJ Head K Alvarez Linera J Angeles Quiroga M Chun Shih P Jung RE Gray matter correlates of fluid, crystallized, and spatial intelligence: Testing the P-FIT model Intelligence 2009 37 124 135 10.1016/j.intell.2008.07.007

[B4] Haier RJ Colom R Schroeder DH Condon CA Tang CY Eaves E Head K Gray matter and intelligence factors: Is there a neuro-g? Intelligence 2009 37 136 144 10.1016/j.intell.2008.10.011

[B5] Haier RJ Stelmack RM Brain imaging studies of personality: The slow revolution In On the Psychobiology of Personality: Essays in Honor of Marvin Zuckerman 2004 Elsevier, Amsterdam 329 340

[B6] DeYoung CG Hirsh JB Shane MS Papademetris X Rajeevan N Gray JR Testing predictions from personality neuroscience: Brain structure and the Big Five Psych Science 2010 21 820 828 10.1177/0956797610370159 PMC3049165 20435951

[B7] Holland JL Making Vocational Choices: A Theory of Vocational Personalities and Work Environments 1997 3 Psychological Assessment Resources, Inc, Lutz, FL

[B8] Kelso GI Holland JL Gottfredson GD The relation of self-reported competencies to aptitude test scores J Vocat Behav 1977 10 99 103 10.1016/0001-8791(77)90046-X

[B9] Larson LM Rottinghaus PJ Borgen FH Meta-analyses of Big Six Interests and Big Five personality factors J Vocat Behav 2002 61 217 239 10.1006/jvbe.2001.1854

[B10] Holland JL Fritzsche BA Powell AB The Self-Directed Search (SDS) technical manual 1994 1994 Psychological Assessment Resources, Inc, Lutz, FL

[B11] Haier RJ Schroeder DH Tang CY Head K Colom R Gray matter correlates of cognitive ability tests used for vocational guidance DOI:dx.doi.org BMC Research Notes 2010 3 1 206 10.1186/1756-0500-3-206 20649948 PMC2917438

[B12] Tang CY Eaves EL Ng JC Carpenter DM Kanellopoulou I Mai X Schroeder DH Condon CA Colom R Haier RJ Brain networks for working memory and factors of intelligence assessed in males and females with fMRI and DTI Intelligence 2010 38 293 303 10.1016/j.intell.2010.03.003

[B13] Carson AD The integration of interests, aptitudes, and personality traits: A test of Lowman’s matrix J Career Assessmt 1998 6 83 105 10.1177/106907279800600106

[B14] Carson AD The relation of self-reported abilities to aptitude test scores: a replication and extension J Vocat Behav 1998 53 353 371 10.1006/jvbe.1998.1641

[B15] Holland JL Self-Directed Search Form R 1994 4 Psychological Assessment Resources, Inc, Lutz, FL

[B16] Ashburner J Friston KJ Voxel-based morphometry–the methods Neuroimage 2000 11 805 821 10.1006/nimg.2000.0582 10860804

[B17] Mazziotta JC Toga AW Tissue Probability Map Provided by the International Consortium for Brain Mapping (T1 452 Atlas) [http://www.loni.ucla.edu/Atlases/Atlas_Detail.jsp?atlas_id=6]

[B18] Good CD Johnsrude IS Ashburner J Henson RN Friston KJ Frackowiak RS A voxel-based morphometric study of ageing in 465 normal adult human brains Neuroimage 2001 14 21 36 10.1006/nimg.2001.0786 11525331

[B19] Horn JL McArdle JJ, Woodcock RW A basis for research on age differences in cognitive abilities In Human Cognitive Abilities in Theory and Practice 1998 Lawrence Erlbaum, Mahwah, NJ 57 92

