# Supplementary figures and images for: Regional gray matter correlates of vocational interests
Source: BMC Res Notes. 2012 May 16;5:242. doi: 10.1186/1756-0500-5-242 (PMC3476449; doi:10.1186/1756-0500-5-242)

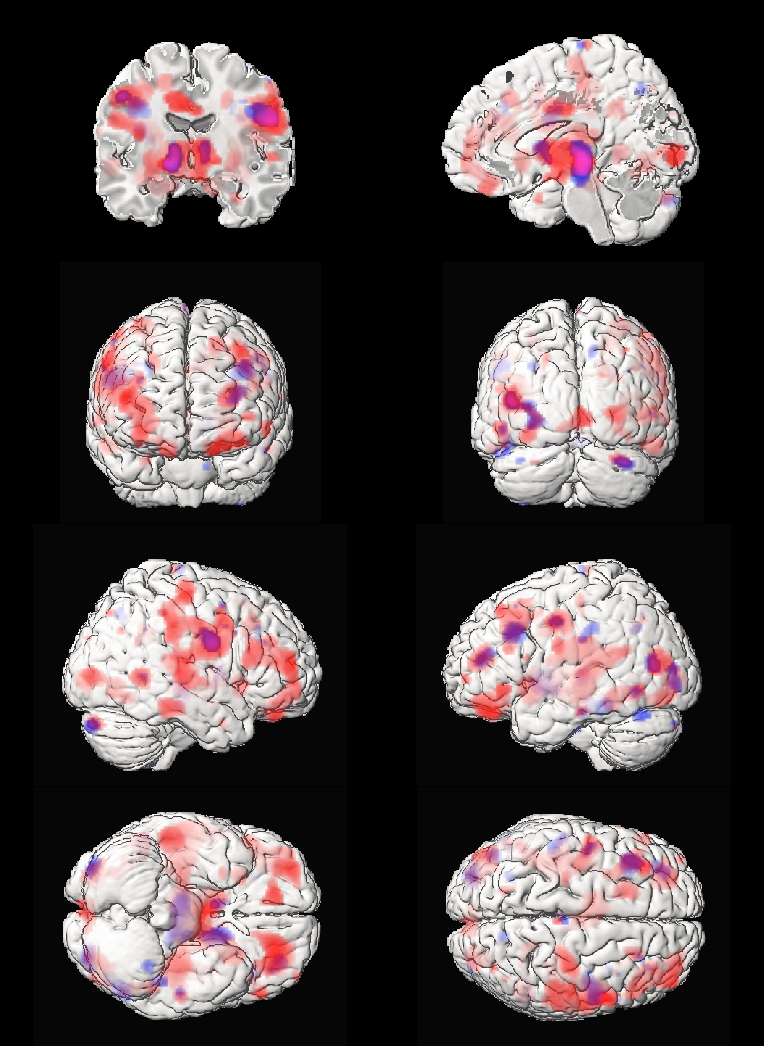

Supplement: Additional file 1 — Figure S1. Gray matter correlates of Investigative interest scores (red) and JOCRF g scores (blue), both shown at p < .025. Overlap is purple. (JPEG 455 kb) [file 1756-0500-5-242-S1.jpeg]

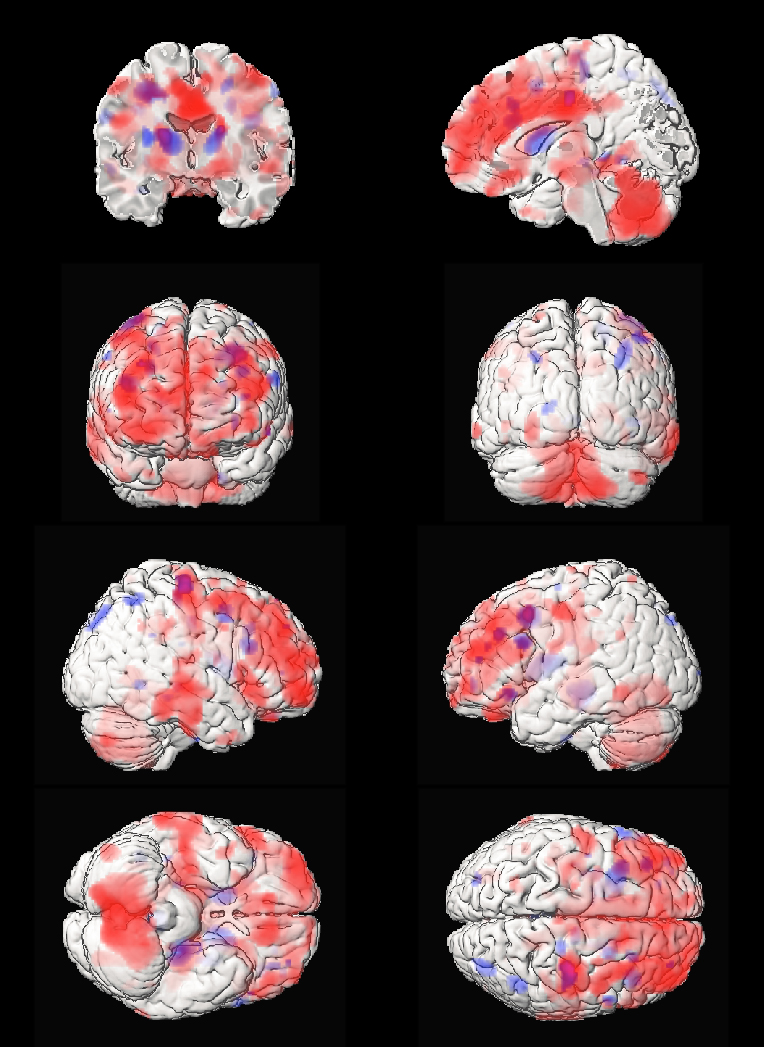

Supplement: Additional file 2 — Figure S2. Gray matter correlates of Realistic interest scores (red) and JOCRF Spatial scores (blue), both shown at p < .025. Overlap is purple. (JPEG 460 kb) [file 1756-0500-5-242-S2.jpeg]
